# An update on risk communication in the Arctic

**DOI:** 10.3402/ijch.v75.33822

**Published:** 2016-12-13

**Authors:** Eva-Maria Krümmel, Andrew Gilman

**Affiliations:** 1 Inuit Circumpolar Council, Ottawa, Canada; 2 Sustainable Solutions International, Parksville, BC, Canada

**Keywords:** contaminants, circumpolar, Indigenous peoples, Inuit, traditional diet, country foods

## Abstract

**Background:**

Arctic residents can be exposed to a wide range of contaminants through consumption of traditional (country) foods (i.e. food from wild animals and plants that are hunted, caught or collected locally in the Arctic). Yet these foods provide excellent nutrition, promote social cohesion, meet some spiritual needs for connectedness to the land and water, reinforce cultural ties, are economically important and promote overall good health for many. The risk and benefit balance associated with the consumption of traditional Arctic foods is complicated to communicate and has been referred to as the “Arctic Dilemma”. This article gives an update on health risk communication in the Arctic region. It briefly summarizes some research on risk communication methodologies as well as approaches to an evaluation of the outcomes of risk communication initiatives. It provides information on specific initiatives in several Arctic countries, and particularly those that were directed at Indigenous populations. This article also summarizes some international versus local risk communication activities and the complexity of developing and delivering messages designed for different audiences. Finally, the potential application of social media for risk communication and a summary of “best practices” based on published literature and a survey of Inuit in a few Arctic countries are described.

**Conclusion:**

Several of the risk communication initiatives portrayed in this article indicate that there is only limited awareness of the outcome of risk communication messages. In some cases, risk communication efforts appear to have been successful, at least when effectiveness is measured in an indirect way, for example, by lower contaminant levels. However, due to missing effectiveness evaluation studies, uncertainty remains as to whether a specific risk communication method was successful and could be clearly linked to behavioural changes that resulted in decreased contaminant exposure.

Accumulating evidence of contaminants in traditional (country) food prompted a research team to undertake a small study in the Inuit community of Qikiqtarjuaq (Broughton Island) in Nunavut, Canada (1). The communication of the Broughton Island results, that is, that breast milk had high levels of polychlorinated biphenyls (PCBs), caused alarm and confusion in communities (2). It was reported that many people ceased to eat traditional foods altogether, which led to more immediate health problems and undermined the nutritional benefits of a diet consisting of traditional food. Further contaminants research found that blood and breast milk of Inuit women from the Hudson Bay area also had elevated levels of persistent organic pollutants (POPs) (3). PCB concentrations in the blood of many individuals living in the Arctic, including two-thirds of those under 15 years of age, were above 5 µg/L, which was considered to be an exceedance of tolerable blood levels at the time (4). The experience during the communication of the Broughton Island study results highlighted the need for work on contaminants and risk communication to be undertaken concurrently as well as the necessity of including Indigenous representation when addressing health concerns. A brief history of the evolution of health risk communication in Canada can be found in the Arctic Monitoring and Assessment Programme (AMAP) Human Health Assessment (5).

Health risk communication involves messages and advice designed to reduce harm and maintain and improve health, delivered in a culturally and socially respectfulmanner. Risk communication has as its foundation good health risk assessment, that is, evaluation of all the available science including epidemiological evidence, animal studies and a determination of “safe levels” of exposure. A more detailed description of the risk assessment process, including risk communication, has been provided by Odland et al. (6).

The health risk communication process requires communication and information sharing to take place between risk assessors, risk managers, the local community, news media and interest groups (6) and can be very complicated, especially in the Arctic region. For example, the development of risk communication messages needs to take regional and cultural differences in diet into account, as well as the fact that multiple food types are consumed, which contain mixtures of contaminants. Important aspects of delivering messages include interactions between the sender (e.g. health officials) and the receiver (e.g. people in a community) of risk communication.

However, more effort has been invested in the identification, monitoring and assessment of effects of human exposure to environmental contaminants compared to learning how best to inform or influence public decision-making to protect health and culture in the Arctic.

## Approaches to risk communication

There are many theories on how behaviours can be influenced to reduce risks to health. There are also models which describe how risk communication can be presented (framed) for greater uptake. These theories and models when combined with the specific challenges of communicating health-based messages may be instructive for developing and evaluating risk communication strategies in the Arctic.

Over 25 years ago, Covello and Allen (7) provided the following seven cardinal rules of risk communication, most of which appear to be applicable even today:

- Accept and involve the public as a legitimate partner
- Plan carefully and evaluate efforts
- Listen to the public's specific concerns
- Be honest, frank and open
- Coordinate and collaborate with other credible sources
- Meet the needs of the media
- Speak clearly and with compassion.

Different risk communication theories offered by these and other authors (8–13) address several key considerations that influence the uptake and effectiveness of risk communication messages. Effectiveness of a risk communication message is often modulated by personal beliefs and an understanding of the benefits of changing a behaviour. A concern that the consumption behaviour poses a personal health threat or a threat to people who are important to the individual is a significant consideration when deciding to change a behaviour. This includes a view that the cost of changing behaviour outweighs the cost of adopting the behaviour (e.g. in social, financial and conformity terms).

Weinstein (14) argued that decisions made by individuals about changing their behaviour are based on their common understanding of information from a wide range of sources. In addition, there are considerable differences in what individuals perceive as “correct” information. Where there are differences which matter in individual perceptions versus scientific fact, information and strategies that update perceptions may add significantly to the effectiveness of an advisory.

Sandman et al. (15) examined how a risk is perceived depending on where it is located on a gradient of risks (risk ladder) well known by the population. They concluded that the formats used in the risk communication (framing) were significant elements for designing effective advice. Connelly and Knuth (16) studied framing with respect to fish consumption advisories and concluded that a diversity of approaches is necessary for effective communication of risk, for example, written material in a good and understandable language (not to be confused with simple language), videos, signs, maps, symbols and/or interpersonal contacts. Good health risk communication is best formed within an interdisciplinary frame, and expertise is required in various fields such as programme planning, evaluation, communications theory, marketing and public health (17).

## Evaluating the effectiveness of risk communication strategies

The notion that evaluation of effectiveness is an important component of any risk communication campaigns is not new. Several authors stated the need for pretesting messages and/or evaluating communication efforts (7,17). Coffman (13) offered different evaluation approaches that can be considered at different stages of the risk communication.

For most Arctic risk communication initiatives, there appears to have been very little pretesting of risk messages. In the last 10 years, only a few evaluative exercises have been reported, which contribute to an understanding of successes and challenges related to the delivery of risk information. Furgal et al. (18) described how poor risk communication can lead to fear, confusion, undesirable changes in dietary behaviour and traditional lifestyles, and impacts on society, economy and health in Indigenous communities in the Canadian Arctic. Myers and Furgal (19) reported that women of childbearing age in four communities in Canada did not appear to have understood, remembered or acted upon the messages on contaminants in traditional foods and the potential health impacts for the developing foetus. It is difficult to assess ifthe target audience did not understand the messages or if they did not receive, register or remember the message. There is also the possibility that they could not comply with the message due to extenuating circumstances (i.e. food insecurity).

The difference in knowledge systems between the Indigenous populations of the Arctic and the primarily non-Indigenous populations outside the Arctic can also hinder a full understanding of risk communication messages on environmental contaminants. While efforts have been made in some regions (e.g. Nunavut) to develop material that has Inuktitut terminology for contaminants, for some Indigenous populations, it is difficult to understand the concept of “invisible” risks such as contaminants, which have no equivalent word in their languages, may not have a direct effect on health in the immediate future or may be considered a lower priority compared to other issues within Indigenous societies (2,18). It cannot be assumed that the views and concerns on risks held by researchers and government bodies outside the Arctic are shared by residents of the North (19). Perceptions of contaminant risk must be linked with a population's relationships with, and views of, traditional foods to develop effective risk communication messages that result in individual responses to reduce risk (20).

Acceptance of information pertaining to levels of contaminants, potential risks and consumption guidelines for traditional foods is highly dependent on trust, that is, where there is little trust between an Inuit community and a government agency or public health office, there is less likelihood that the community will believe the source, the message or take up the advice (21). More complete information on the benefits of consuming traditional foods and some of the issues associated with consuming store-bought foods, which contain high levels of carbohydrates, especially sweeteners, and unhealthy fats, may better enable Arctic residents to make appropriate choices about their diets.

Binnington et al. (22) examined the effectiveness of maternal fish advisories using a mathematical time-variant mechanistic model (CoZMoMAN). While they found that dietary fish substitution reduced maternal exposure for substances such as PCBs if the advice was followed for at least 5 years, compliance with an advisory as structured in their model was essentially of no value if only followed for a year. These model estimates could be helpful in the future for determining whether both the length and nature of food advisories, designed to protect the foetus and the developing infant from placental and breast milk exposures, respectively, will be effective.

## Recent Arctic-specific experiences in risk communication

The following sections provide some examples of risk communication experience from the Arctic, different strategies and, if available, how effectiveness has been evaluated. General advisories about food and nutrition issued in each of the Arctic countries, such as those on fish consumption, environmental contaminants and pregnancy, are not included here. These general advisories are constantly being updated based on new scientific information on health effects and benefits associated with consuming traditional foods as well as insights in risk communication. Although an effort was made to include information for risk communication activities or experiences for all Arctic countries, there is a lack of information from the European Arctic.

### Alaska, US

Fur seal (*Callorhinus ursinus*) is an important subsistence food for the Aleut of several islands in Alaska and Kamchatka (Russia). Duncan et al. (23) found that 109 of 146 (75%) fur seal placentas were positive for the bacterium *Coxiella burnetii*. In humans, this bacterium can cause the illness known as “Q fever.” Concern about exposure to *C. burnetii* and the safety of consuming fur seal resulted in an inclusive health consultation process with some residents (24).

Public health officials from the State of Alaska, the Alaska Native Tribal Health Consortium and the Centers for Disease Control (CDC) consulted with regional and local tribal health authorities. Based on the consultation, tribal resolutions requested that human serum samples collected from residents (1980–2000) and stored at the Alaska Native Serum Bank be tested for *C. burnetii* antibodies. Analysis by CDC found a seroprevalence of 11–12% (24). For comparison, the seroprevalence of this antibody in the US all-races population is 3% (24). Subsequent health consultations explained that *C. burnetii* exposure occurs primarily through inhalation and not through ingestion and that fur seal was a safe traditional food. CDC offered community blood testing to determine whether there had been any change in exposure to *C. burnetii*, and local health care providers incorporated Q fever in the differential diagnosis for patients with unexplained febrile illnesses (especially prolonged fever and elevated liver enzymes). No locally acquired cases of Q fever have been identified in Alaska (24). This could be due to the symptoms, described above, which are those of community-acquired pneumonia due to a large variety of infectious agents. Q fever has only very recently become known to Alaska health care providers as a zoonotic infection common in an Alaskan animal species.

Owing to the migratory route of northern fur seals, Alaska Natives share this resource with communities in Kamchatka. Considering that rapid environmental change will result in further incidences of zoonotic diseases, improving communication with Russian health officials is seen as an important aspect of the risk evaluation and management process for Aleut health issues, as there are noactive formal relationships with public health officials in Russia. Copies of the public health bulletins developed by the State of Alaska (24) were sent to the Aleut International Association which has formal ties with communities in Kamchatka. No formal analysis on the effectiveness of communication has been performed; however, the project was inclusive with active participation by US federal, state, tribal and local officials. Project opportunities and options were presented, and actions were taken based on local preference and with local permission.

### Canada

#### Risk communication in Nunavik

Between 1993 and 1996, analysis of umbilical cord blood of Inuit newborns found that 7% of the blood lead (Pb) levels were above the blood Pb intervention level (25), and approximately double the concentration of blood Pb levels in the newborns in southern Quebec (26). The likely reason for the higher blood Pb levels was the use of lead shot for hunting.

In 1999, several Nunavik regional entities acted to remove lead shot from use and replace it with steel shot or other alternatives (5). The resulting Regional Coalition of the Banning of lead shot in Nunavik implemented an awareness campaign that included municipal officials and merchants, local radio announcements, articles in various periodicals, and posters and brochures in three languages (Inuktitut, French and English).

Couture et al. (27) reported that blood Pb levels in Inuit from Nunavik had decreased significantly and particularly after the intervention in 1999, but remained higher than in southern populations. It is uncertain whether the changes in Pb levels were only due to the intervention or a general shift in diets away from hunted waterfowl. The availability of lead shot has declined in many stores in Nunavik, but is still available in some places. A survey of hunters in Inukjuak showed that only 31% of respondents were aware of the ban on the use of lead shot. While there is evidence that the concerted intervention in 1999 initiated a positive result (lower blood Pb levels), the effectiveness of the messaging and the role played by the hunters who may have switched to steel shot versus those who prepare the meals so as to exclude lead shot have not been assessed.

Exposure to PCBs and mercury (Hg) in Nunavik is mainly due to the consumption of marine mammal fat, especially beluga (*Delphinapterus leucas*) blubber for PCBs, or marine mammal meat, especially beluga muscle for Hg (28). The Nunavik Child Development Study (NCDS) conducted from 2005 to 2010 found that exposure to contaminants was related to health and developmental effects.

The results of the NCDS led to a shift in risk communication where the public health messages were revised to focus mainly on pregnant women and women of childbearing age, and on how to reduce exposure to Hg and Pb while maintaining an intake of n-3 fatty acids. Since PCB exposures had declined significantly in the population between 1994 and 2001 (29) as well as in the environment, no individual recommendation for reducing exposure to PCB was included because children born recently would be much less exposed than those in the original NCDS cohort. More detailed information was also made available on the Internet for several POPs, Hg and Pb.

The campaign to communicate the results from the NCDS was extensive: it included study participants (parents and children); the general population; employees of the regional health and social services network; midwives; regional, national and international Indigenous organizations; regional contaminant committees; health officials from other Northern regions of Canada; representatives of the Northern Contaminants Program (NCP); Health Canada; and the general public. The communication of results was carefully planned to ensure that information reached to the Nunavik population before it was presented at scientific meetings and in peer-reviewed journal articles. An effectiveness evaluation component is underway.

#### Risk communication in Nunavut

Inuit in Nunavut expressed a desire to have health information of practical relevance so that they could make informed decisions in the face of the rapid changes that are affecting all dimensions of life in their communities. In response to these concerns, a large and complex participatory health research project was developed and undertaken in 25 communities in Nunavut in 2007 and 2008 (30). The goal of the Nunavut Inuit Health Survey (NIHS) was to obtain an overview of the health status and living conditions of Inuit aged 18 and over in Nunavut. The results of the work led to the following key messages related to food and contaminants:

Country foods provide many essential nutrients that can lower the risk of chronic diseases. Most Inuit adults in Nunavut need not be concerned about contaminant-related effects from country food consumption. Generally, the benefits of eating country foods outweigh the risks from contaminant exposure.

Inuit women of child-bearing age who may become pregnant, are planning to get pregnant, or are pregnant should avoid eating ringed seal liver due to its high mercury content. Instead, ringed seal meat is a great and healthy alternative…

Over the course of 2013 and 2014, the NIHS Steering Committee undertook a project aimed at reviewing the efficacy of NIHS contaminants communication, covering three communities in Nunavut and over 1,000 participants. Preliminary results were published in the 2014 NCP Synopsis Report (31), which showed that fewer than half of the people surveyed remembered hearing the riskcommunication messages on avoiding certain traditional foods due to contaminants, while over 80% of participants reported hearing about the benefits of traditional food. One-third of participants stated that they had modified their eating habits after hearing about contaminants in traditional foods. The most popular sources of information were “friends or family,” radio and television. The authors of the study found that responses differed between the three communities, and emphasized the need to conduct evaluations after risk communication activities to ensure that messages were released and received as planned and expected.

## Faroe Islands: dietary advice on consumption of pilot whale

High levels of Hg in meat and organs of pilot whales, which are an important traditional food source for the Faroese, were first reported in 1977 (32). This finding led to the first consumption advisory for the general Faroese population from the Chief Medical Officer to limit the consumption of pilot whale to one meal per week and to completely avoid pilot whale liver and kidney. Since 1980, pregnant women were specifically advised to limit their consumption of pilot whale meat and blubber. In 1989, additional information on high levels of organochlorine contaminants in the blubber of pilot whales led to the consumption advisory that not more than 200 g of whale meat and blubber (each) should be consumed per month and that pilot whale liver and kidney should be avoided completely. In 1998, another advisory followed due to demonstrated effects of Hg and PCB exposure on the health of the foetus and newborns. This advisory focused on adults and, most specifically, on young and pregnant women.

The Faroese body burden of contaminants is still high in comparison with other populations and is associated with adverse health effects (5). In 2008, the Faroese health authority concluded that pilot whales currently exceed limits for acceptable concentrations of toxic contaminants and can no longer be recommended for human consumption (32).

**Fig. 1:**
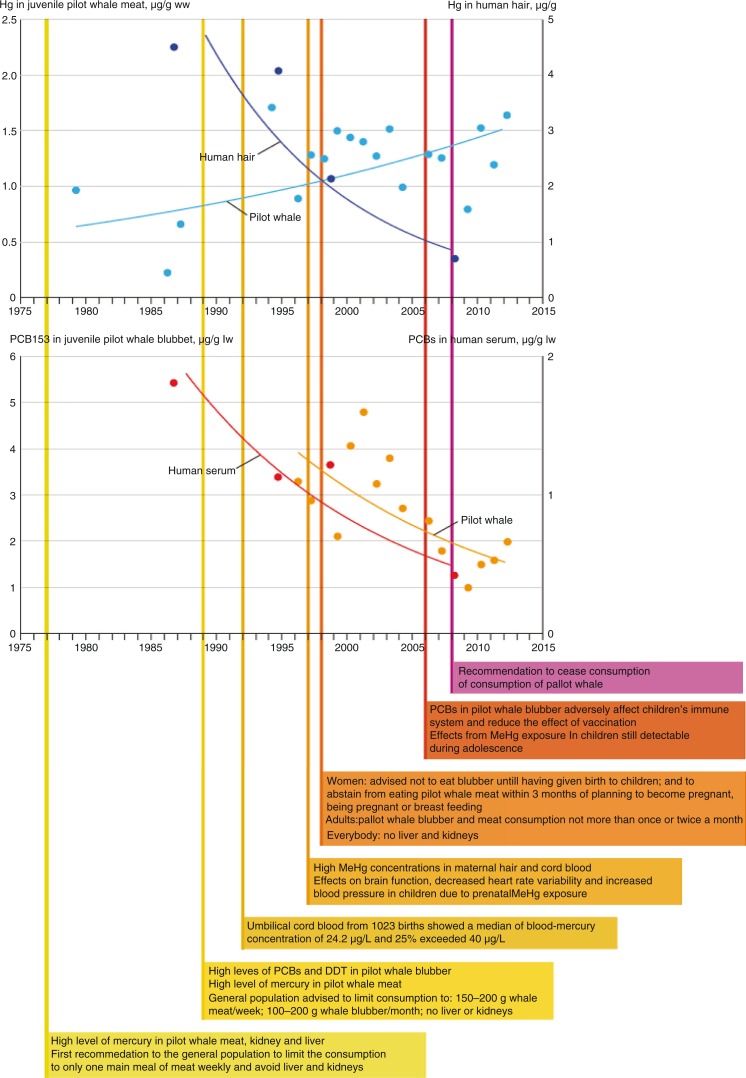
Timeline of risk communication for mercury (Hg) and polychlorinated biphenyls (PCBs) related to pilot whale consumption in the Faroe Islands. Reproduced with permission from AMAP.

In the case of the Faroe Islands, the risk communication efforts appear to have been successful in convincing pregnant women to consume less pilot whale than before (6,32). While Hg levels in pilot whales have not decreased over the last three decades, concentrations in the blood of pregnant women have decreased significantly (Fig. 1). Although a dietary shift can be caused by several factors, it is likely that the risk communication undertaken in the Faroe Islands was the driving force for the decreases in human tissue levels of Hg and PCB for several reasons. For example, associated with the extensive cohort studies that have been ongoing since 1985, risk communication was continuous throughout the years and reached all areas of the islands (6). Further, risk communication messages were always restricted to pilot whale consumption, and several fish species with low contaminant concentrations were available and recommended as alternative dietary choices. However, health effects are still measurable even at these lower levels of exposure (5). In addition, the success of the risk communication efforts and lower levels of contaminants in the Faroese population come at a cost of loss of cultural identity for the Faroese people, who have relied on pilot whales as a staple part of their diet for hundreds of years (32,33).

## Greenland: addressing conflicting evidence about diet and health

A country-wide population survey from 2005 to 2009 on adult Inuit in Greenland found that 78% exceeded 5.8 µg/L, a Hg blood guideline level established by the United States Environmental Protection Agency, and 98% exceeded 5 µg/L blood PCB concentrations (34). At the same time, there was concern about a pronounced increase in overweight individuals and diabetics during the previous 15 years due to the dietary transition from a nutritious traditional diet to a less healthy diet based on store-bought foods.

The Greenland Board of Nutrition is tasked with providing balanced information to the public about contaminants in the traditional marine food diet and general information about a healthy and nutritious diet. Recently, it was determined that obesity plays a greater role in adverse health outcomes than exposure to contaminants (34). As a result, dietary advice to the general Greenlandic population has been revised and it consists of 10 simple recommendations (34), which include eating a variety of foods and to eat local foods, fruit, vegetables, fish, whole grains, etc., daily or often, eat less sugary foods or drinks, and to be physically active for at least an hour per day.

There are also recommendations on physical activities and social aspects of preparing and eating meals. In addition, pregnant and nursing women (a book is provided to all pregnant women), as well as children and young people, are encouraged to continue to eat traditional marine food and to avoid or reduce consumption of older seals, toothed whales, seabirds and polar bear due to high concentrations of contaminants. It is recommended that individuals substitute these foods with lean fish and terrestrial mammals. The specific focus in the recommendation is to promote availability and consumption of more fish that are known to have the lowest contaminant levels. The recommendation is directed at all parts of the society (families, child care centres, schools, elder care facilities, hospitals, etc.) and commerce (fishermen, fish shops, sales and delivery, etc.).

Bjerregaard and Mulvad (34) reported that in 2007 the Greenland Board of Nutrition evaluated the success of an information campaign on dietary recommendations. Their “outcome” evaluation found that 43% of respondents knew about the campaign, mostly from television and brochures, and were familiar with recommendations regarding eating fruit and vegetables and traditional food. However, it was not known whether the same respondents also followed the advice.

## Russia: POPs and metals in Chukotka

The AMAP Russian Arctic Persistent Toxic Substances (PTS) study examined contaminants in food samples from Chukotka and undertook dietary surveys of the Indigenous people living in inland Kanchalan and the coastal Uelen settlements (35). It was found that some marine wildlife exceeded Russian food safety limits. For example, livers of whales, walruses and seals exceeded the food safety limits for cadmium (Cd) by 5 to 15 times, and all species of seals exceeded limits for Hg by 3 to 100 times. Kidney and liver of walruses (*Odobenus rosmarus*) and grey whales (*Eschrichtius robustus*) exceeded the safety limits for Hg by two to four times. It was also suggested that houses and food containers were a source of POPs (e.g. through using insecticides in the home and preparing food and alcohol in contaminated containers). As a result, some prepared food items (such as fermented walrus meat) and homemade alcohol were highly polluted with PCBs and dichlorodiphenyltrichloroethane (DDT).

Based on the PTS study findings, consumption restrictions were recommended for several species and tissues, and several risk communication measures were implemented to reduce contaminant exposure (36). The Ministry of Health Care and Social Development of the Russian Federation approved systematic population health inspections, contaminant waste clean-up projects and training sessions. Risk communication initiatives included brochures, a film, community meetings, non-technical summaries of the PTS, a school education programme and broad media coverage of the issues. The risk communication initiatives were prepared and distributed to local Indigenous communities; school teachers and pupils; managers of local administrative, maintenance, health and sanitary services; and other groups involved in decision-making on a wide range of quality of life issues.

Dudarev (36) reported that the comprehensive study approach in Chukotka and the thorough awareness campaign were very successful in producing desired “outcomes,” that is, implementation exercises and contaminant exposure reduction measures. However, to date there has been no follow-up study to investigate whether the measures taken have caused a desired “impact,” that is, a decrease in contaminant exposure.

## Circumpolar Inuit perspectives

The Inuit Circumpolar Council (ICC) recently completed a report to address circumpolar perspectives on risk communication: views from Alaska, Canada, Chukotka and Greenland (21). The methodologies of the report and how information was obtained, as well as more details on findings, are summarized in the AMAP Human Health Assessment (5). The majority of views reflected Inuit perspectives. Because it was found that perspectives can vary widely between regions or even within one region, the findings should not be extrapolated to be considered representative of all Indigenous peoples in the Arctic.

Most regions reported that risk communication varied by issues and communities. In two countries there was no national strategic approach to risk communication for their Arctic region (Alaska and Chukotka), while in two other countries (Canada and Greenland) there was an integrated national approach for risk communication development and dissemination.

With regard to the effectiveness of risk communication activities, the responses from the recipients of the risk messages appear consistent in their view that there are few behavioural changes and few studies which indicate that the messages were effective (5,21).

## International risk communication experiences related to the Arctic

Risk communications on contaminants are of importance on the international stage, for instance, during international scientific and policy meetings related to contaminants and/or to support negotiations for multinational environmental agreements which may impact the Arctic. However, when sharing concerns about the risks posed by contaminants to the health of Arctic Indigenous peoples, specific research findings and risk reduction messages can be a double-edged sword. Although some specific risk communication messages are intended for the protection of a local community or regional population from possible adverse health effects of contaminants found in parts of their diet, these same public health messages can be spread to other communities, regions or countries through the availability of global media, and particularly via the Internet. Rapid dispersal of messages meant for one group or location can create anxiety and confusion in other areas where the scientific information or the advisory does not apply, or for which it was not intended.

For example, an international conference in 2011 on Climate Change and Pollution included a presentation from the Faroe Islands about the inhibitory effects of PCBs on the efficacy of vaccinations (37). The conference presentation also explained that the findings of the research led to a health advisory in the Faroe Islands suggesting that local communities refrain from eating pilot whale (see above). Subsequent media reporting by an Arctic newspaper that the same recommendation would be valid for the consumption of other toothed whales in other areas in the Arctic, including Canada, led to concerns there that the reports would discourage Canadian Inuit from eating their traditional foods.

The Faroe Island example underlines an inherent obstacle in sharing risk communication messages ininternational fora; news about research results and risk reduction strategies may be extrapolated to other regions, regardless of the relevance or validity of the advice. This can be a challenge for the development of trustworthy risk communication messages in other regions and for specific groups in specific locations where contaminant levels and food intakes have been measured and evaluated and have not been found to pose a similar risk.

The ICC had similar experiences during the negotiations for legally binding instruments for implementing global action on POPs and Hg (Stockholm Convention and Minamata Convention, respectively).

For the POPs negotiations, several Canadian Indigenous partners formed the coalition Canadian Arctic Indigenous Peoples against POPs to advocate for consensus and urgency (38). The international messages throughout the POPs negotiations made their way back to Canada and into the Arctic regions through international media, and seemed to contradict the messages developed for local constituents at home (19).

Contrary to messages during the POPs negotiations, ICC interventions during the Hg negotiations 2010–2013 were able to draw directly from Canadian regional consumption advisories (see above) to highlight impacts of atmospheric Hg emissions on Inuit in the Canadian Arctic. However, while ICC carefully worded messages in its press releases, changes in wording by subsequent media reporting still caused local concern (5). While ICC referred to food advisories due to high Hg levels in some traditional food items, the media just took a broad-brush approach, stating “Inuit consume mercury when they eat country food.” Such generalized messages may cause anxiety and uncertainty in Northern regions, where the overall dietary advice given by health officials is that traditional food is healthy and should be consumed.

Overall, the experience of risk communication on the international scale shows that it is not desirable for one source to use messages with significantly different content for different audiences, since this message can reach a non-target audience through global media sources such as the Internet. At the same time, it is likely that different messages are transmitted by different (global) sources and reach local non-target audiences, which can cause confusion in local populations. Therefore, continuous communication is required locally to reinforce the validity of messages to a local audience and prevent confusion through non-local information sources that may not be valid for a specific local audience.

## Application of social media for risk communication in the Arctic

Use of social media, that is, “various electronic tools, technologies, and applications that facilitate interactive communication and content exchange, enabling the user to move back and forth easily between the roles of audience and content producers” (39), is becoming a potent tool for all forms of communication, including health risk communication. However, the use of social media for health risk communication differs from more commonly used approaches in that the former involves a multiway communication (between the originator and social media users) whereas the latter tends to rely more on the one-way flow of information (usually as a top-down approach from health experts to the target group). Using social media for a dynamic exchange of information rather than a more traditional passive communication facilitates the development of a greater understanding of how health risk messages can elicit different responses based on who delivers the messages, how they are delivered and how the public actively processes the information (40).

Social media can be used to meet the objective of providing health information in an accessible, timely manner, and can be used as a strategy to monitor the perceptions, reception and understanding of the message and contributions to the conversations (41,42).

### Social media for health risk communications: forms, practices and effectiveness

The form of social media used within a health communication strategy largely depends on the goals of the strategy, whether it is message dissemination and/or public engagement (43).

*One-way electronic-media-based communication* (email and many websites) is a form of information dissemination that is often more accessible now compared with traditional media forms (e.g. radio, television, letters, brochures and newspapers). Nevertheless, the accessibility of different forms of media and communication sources remains audience dependent. Internet-based content with graphics, colour, text and audio can be updated rapidly but is still not fully enabled for discussion or rapid group interaction. YouTube is one of the best-known content communities used for video sharing (44).

*Two-way communication* represents perhaps the greatest shift in risk messaging communication possibilities, especially when combined with access to information available online. Two-way media allow for an exchange of comments, feedback and greater clarity in response to feedback. They are open to discussions on the disparity of views arising from the factors influencing risk perception and behaviour in Northern communities, such as cultural, social and demographic factors (18,42).

*Social networking sites* are websites used for building virtual communities, organizations or personal networks, allowing users to connect and socialize online (44–46). Social networking sites are another way for health messages or campaigns to be advertised and targeted to appropriate groups. However, people would need to be linked to the discussion group or organizations’ web pages in order to receive the health messages.

### Social media as a tool and opportunity for risk communication in the Arctic

Social media as a tool for health risk communications can provide opportunities in the circumpolar North to enhance risk communication strategies. For communities with few resources to put towards the communication of health risks, social media could help overcome cost barriers as several social media tools offer a relatively cost-effective means of communication (41), especially for reaching out in-person to remote communities. Two-way communication and community engagement via social media is one means to build mutual trust between public health officials and community members; this is especially critical in the North where there is much health research underway related to contaminants and scientists and health officials are unable to make sufficiently frequent visits to communities to provide updates.

Health messages being delivered by Northerners to Northerners can provide optimal trust in the message. For example, for the NCDS described earlier, the Nunavik Assistant Director of Public Health was chosen as the spokesperson for the YouTube capsules. As an Inuk, she was considered by the communities as both a representative of the people and a credible professional (46). In this case, the YouTube capsules were included in an effort to increase the effectiveness of messaging to youth and young women of childbearing age. Videos were purposefully kept to less than 2 min and just provided the basic results of the study (46). Longer video clips may be useful for other target audiences for which more background information to the study would be valuable.

Not all communities in the Arctic have reliable access to broadband Internet, an essential component for effective social media messaging. Among the circumpolar countries, Iceland, Alaska and Norway have the highest percentages of population using the Internet, followed by Sweden, Finland and Northern Canada (47). Greenland has over 90% of its population connected to Internet (48). Arctic Russia has the lowest percentage of the population using the Internet. In those countries and regions with limited Internet availability, radio or TV may continue to be a better communication tool for widespread distribution of risk communication messages.

Three confounding factors make the evaluation of risk messaging in social media fora difficult: a general lack of understanding of how social connections impede or validate/support behaviour change, whether or not to have confidence in forum moderators (in terms of how they might direct or influence the conversation) and knowing who might have joined a social networking group and why (e.g. their motivation and bias) (41,49). These factors need to be taken into account when considering the use of social media tools for undertaking research on disseminating risk messages, evaluating public perceptions of messages or gathering research data on effectiveness of messaging.

### Considerations for social media use in Arctic risk communications

There are several Arctic-specific considerations which would enhance risk communication through social media; many are common to the traditional one-way communication forms:

- Preservation of Indigenous language in an online world that works primarily in English
- Risk message text which can be combined with audio, video and/or in-person communication and community workshops
- Establishing trustworthy and credible channels and sources for the social media tools available
- Monitoring and managing the communications online continuously (see 40)
- Avoiding anxiety and fear by providing correct and clear information on contaminants in traditional foods and the benefits of these foods
- Directing health messages to the correct target populations and consulting broadly on the messages prior to issuing a risk message
- Streamlining message approval processes to enable real-time communications (see 42)
- Avoiding information overload
- Enhancing staff knowledge of social media tools and promoting technological/IT capacity (see 40)
- Improving the availability of technical infrastructure and the Internet access/bandwidth to enable reliable use of some social media tools
- Recognizing that social media tools may not be as effective for reaching certain demographic groups, for example, seniors.

There is very little published literature on the impact of social media on vulnerable populations. The social media practices of community organizations have yet to be formally evaluated (50). Therefore, given the fairly recent introduction of social media as a tool for risk communication, particularly in the North, it is difficult to say whether such campaigns are or will be effective at reaching target audiences. Research aimed at examining the effectiveness of social media campaigns will assist health communicators in the future use of these tools.

### Optimizing risk communication in the Arctic

Several authors have noted best practices for risk communication with Indigenous populations. They point to the need of understanding the target population and culture (51), and especially those groups that are most vulnerable to the impacts of the exposure event. For environmental contaminants, those most at risk are elders, women of childbearing age, infants and children, residents with chronicdiseases and those on medications that affect the immune system. Other authors highlight the importance of trust (51,52) and the role of Indigenous communities and Indigenous knowledge in risk communication (53). Friendship and Furgal (53) proposed a set of common guiding principles rather than a prescriptive rigid framework for bringing people, different cultures and knowledge systems together. The ICC survey of Inuit in Alaska, Canada, Russia (Chukotka) and Greenland has also provided information about best practices for risk communication, which include the importance of Inuit participation in creating the messages; good relationships, communication and trust between all involved entities; and positive messages (5,21).

Consideration could be given to the following points when using and evaluating social media tools for risk communication in the Arctic:

- Provide messages in multiple formats, that is, use social media tools alongside more traditional forms of risk communication.
- Adopt low-risk social media tools first, that is, those which also allow a good entry point into the use of social media, and then expand into other tools.
- Make strategic choices and understand the level of effort needed to develop and maintain the social media tools selected.
- Understand the target group and their preferred social media sites; the most effective messages are those tailored to specific cultural or demographic groups. Choose communication tools that would enhance the receipt of information, for example, consider using video or audio tools for communities or target groups with low literacy and computer capability, consider short clips for youth who may be used to shorter bursts of information.
- Develop social media messages and tools in collaboration with the communities they are designed to assist.
- Make it easy for people to share the health messages by using social media tools with sharing features that can be used by others on their organizational or personal websites or social networking pages.
- Evaluate the social media communication efforts throughout the full lifecycle of the communication campaign.
- Acquire feedback from community members to gather information on the effectiveness of the messages and social media tools. Feedback from the community can also help with message adaptation and can help build relationships and engagement with communities.
- Use a flexible framework to allow the risk communication strategy to move towards the most useful social media tools.

## Conclusions

Modern advisories focus on changing behaviour patterns by recommending an overall healthy diet and consumption of the safest (least contaminated) traditional (country) foods. Their overall objective is better health and social well-being. However, research on health risk communication and the evaluation of its effectiveness in the Arctic has been slow to develop, and evaluations of the outcomes or impacts of risk communication activities in the Arctic are rare. While some authors have concluded that there was a successful outcome because human tissue levels declined after communication of a contaminant intake advisory, other factors unrelated to the advisory may be responsible for the declines reported.

Risk messages have been effective in global negotiations related to contaminants (both the Stockholm and Minamata Conventions have been successfully launched and mention the particular vulnerability of the Arctic and Indigenous communities). However, the same messages have sometimes resulted in confusion in some local areas where the message was received through the mainstream media and seemed to conflict with local advice.

Social media tools can be a way to overcome some of the obstacles to communicating in the North by using appropriate language and literacy, enhancing the reach to remote communities or particular target groups (e.g. youth) and engaging the communities in two-way communications without the physical presence of a health communicator. Health communicators and several Indigenous organizations and governments targeting Indigenous people in the circumpolar North have begun to incorporate social media tools into their communication strategies online.

A diversity of approaches is necessary for effective communication of risk, for example, tone, presentation of information, reading level, balanced messages, a variety of ways to reduce risk and messages targeted at particular groups. Optimal practices for risk communication in the Arctic include the following aspects:

- Full awareness of the most vulnerable groups in the community
- Involvement of Arctic Indigenous peoples in research and communication
- Clear and consistent messages
- Balanced information delivered by trusted sources
- Multipronged approach using a variety of media realistic for the region
- Adapted use of social media
- Communicating risk more frequently and for sustained periods (years)
- Workshops with all stakeholders (scientists, communicators and locals)
- Evaluation of all risk communication before, during and after the risk communication period.

Overall, risk communication is not a solution to the Arctic contaminant issue. National and international efforts are needed to reduce or eliminate contaminant levels in the Arctic, including supporting and ratifying global agreements to regulate contaminants, such as the Stockholm Convention on POPs and the Minamata Convention on Mercury.
